# Fundamentals of Breast Implant Illness and Device Imaging

**DOI:** 10.1155/2022/4155530

**Published:** 2022-08-13

**Authors:** Eduardo de Faria Castro Fleury, Arthur E. Brawer

**Affiliations:** ^1^Centro Universitário São Camilo, Medicina, São Paulo, Brazil; ^2^Drexel University, Philadelphia, PA, USA; ^3^Robert Wood Johnson, New Brunswick, NJ, USA

## Abstract

The past six decades of silicone breast implant history encompass manufacturing secrecy, regulatory laxity, inadequate informed consent, clever advertising, overly simplistic research methodology, diverse and controversial opinions, changing social patterns, safety issues, information ambiguity, speculation, and deception. This review addresses the verifiable clinical, radiological, and pathological aspects of these devices, particularly with regard to silicone bleeding. This information can favorably assist practitioners and radiologists facing diagnostic challenges encountered in patients with silicone breast implants.

## 1. Introduction

The silicone implants' history involves advertisements, controversies, behavioral changes, speculations, aesthetic standards, safety, trends, and scandals. The ambiguity of information has always been present in the implants' short life.

It is estimated that augmentation mammoplasty accounts for roughly 15% of all plastic surgery procedures worldwide [1]. Plastic surgeons routinely use silicone gel-filled breast implants for aesthetic and repair purposes, and they repeatedly emphasize recipients' physical and emotional satisfaction as justification for their use. In the latter half of the 1980s and the early 1990s, escalating reports of device rupture and allegations of device-related systemic ailments began to surface in hundreds of thousands of recipients [2]. Researchers proposed controversial autoimmune causation theories, prompting proponents and naysayers to seek the judgment of scientific panels. In the latter half of the 1990s, three independent panels refuted the autoimmune theories, leading to the erroneous perception that silicone-induced systemic disease had been permanently laid to rest [3].

The groundwork was cemented for an inevitable recurrence of this previous public health debacle. Over the past eight years, hundreds of thousands of new ailing breast implant recipients in North America and South America have resurrected allegations of device-related systemic ailments [4]. During this same time interval, many plastic surgeons in the USA have become more sympathetic to these complaints. They are now permanently explanting more than 20,000 of these women each year (with subsequent improvement and/or resolution of their physical ailments). These events have paralleled the appearance of a second device-related problem, namely the recognition of an increased risk of breast implant-associated anaplastic large cell lymphoma (BIA-ALCL) in the capsule surrounding the implant.

Two complementary causation concepts exist regarding the T cell malignant transformation seen in BIA-ALCL: (a) chronic antigenic stimulation caused by textured silicone devices and their bacterial biofilms and (b) biochemical disruptions caused by degradation molecules of silicone gel [5]. Likewise, two causation concepts exist for breast implant illness (BII); some investigators have resurrected faulty autoimmune theories in the form of autoinflammatory syndrome induced by adjuvants (ASIA), while others have emphasized widespread biochemical disruptions caused by silicone gel degradation products. After over one-third century of controversy and regulatory laxity, safety issues are finally being properly addressed [6–10].

This review addresses the clinical, radiological, and pathological aspects of silicone bleeding. It discusses the clinical application of this knowledge and the diagnostic challenge in managing patients with silicone implants.

## 2. Composition of Silicone Gel-Filled Implants

Silicones belong to a class of substances called organosiloxanes, all containing artificial silicon-carbon bonds not naturally found in any living organisms on Earth. Over the past 80 years, more than 60,000 organosiloxane compounds have been synthesized for commercial uses. Silicone gel-filled breast implants are composed of organosiloxane polymers, where longer polymer chain length (catalyzed by the addition of heavy metals) confers increasing viscosity. These devices' elastomer shells are also composed of polymers with silicon dioxide (silica) added to form a solid envelope [11]. The original gel-filled devices of the 1970s, 1980s, and early 1990s contained a mixture of small molecules that bled through an intact shell from day one and microdispersed to distant body areas. More recent generations of implants from the past 16 years contain cohesive gel, characterized by higher molecular weight polymers with “stable webs.” [6] However, bacterial biofilms around and within these devices cause gel degradation to smaller molecules, resulting in the same gel bleedingthrough an intact shell (along with the same heavy metal dispersion).

Over time, the implant elastomer shell degrades, increasing its permeability while releasing free silica. Silica has a long and sordid history of causing human misery. In light of the information above, one can readily appreciate that saline implants are also potentially harmful [6, 8, 10].

## 3. Host Reaction to Silicone

The numerous favorable physical properties of organosiloxanes (e.g., their high thermal stability, low thermal conductivity, and resistance to attack by oxygen and/or UV rays) led to erroneous conclusions by chemists that silicone devices would be chemically and biologically inert when inserted into humans. These scientists did not anticipate that when something foreign is inserted into the body, the body's initial response is to destroy it. If that effort is impeded, the body will try to excrete it. If that effort also fails, the body will attempt to isolate it [12, 13].

A fibrous capsule is formed around the device following a breast implant procedure. The fibrous capsule consists of dense fibrosis, also having an inner line of pseudosynovia with histiocytes. As a result of the protective function, the blood supply to the intracapsular environment is restricted. In addition to histiocytes, there are lymphocytes, mast cells, and fibroblasts [14].

Over time, when there is silicone surface degradation or when there is silicone leakage from the intact implant shell, silicone corpuscles encounter the fibrous capsule. At this point, the macrophage phagocytes the silicone resulting in a foamy histiocyte. However, this phagocytosis of the foreign body is frustrated, and the material is eliminated after cell apoptosis, making the process vicious. There is then the activation of the type II inflammatory process with a predominance of recruitment of T lymphocytes, especially those in the CD4 lineage [15, 16].

**Figure 1:**
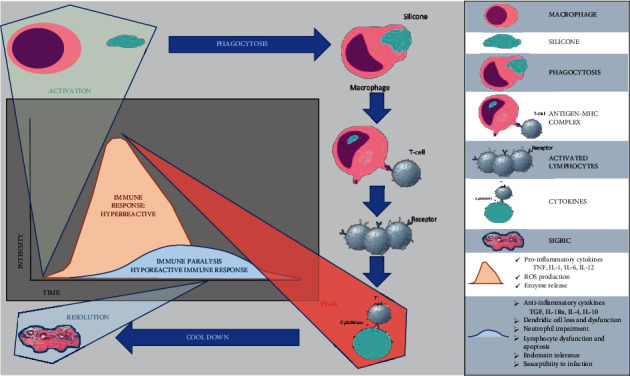
Flowchart demonstrating the silicone-induced granuloma formation flowchart in the fibrous capsule microenvironment. The macrophage phagocytes the free silicone in contact with the fibrous capsule, forming a macrophage antigen complex (MAC) and activating T lymphocytes by MAC. Silicone phagocytosis is frustrated, leading to apoptosis of the macrophage and release of the silicone corpuscles. Activated lymphocytes will release cytokines against the silicone. The process is usually self-limiting and results in granuloma (SIGBIC) formation that is the silicone disease marker.

The inflammatory process is self-regulated and divided into phases: the inflammatory phase, the peak, and regression. When triggered, its intensity can be intervened with, preventing the peak from reaching the inflammatory phase, which is always associated with exudate. When the process is cooled down, a cicatricial granuloma may be observed. Sometimes, when the process is eternalized, negative dysregulation of T lymphocyte activation can occur. In this phase, there is the recruitment of increasingly younger T cells due to high consumption; usually, the immunohistochemical study shows an increase in the expression of CD30 in membranes, inferring with cell proliferation [14] (Figure 1).

**Figure 2:**
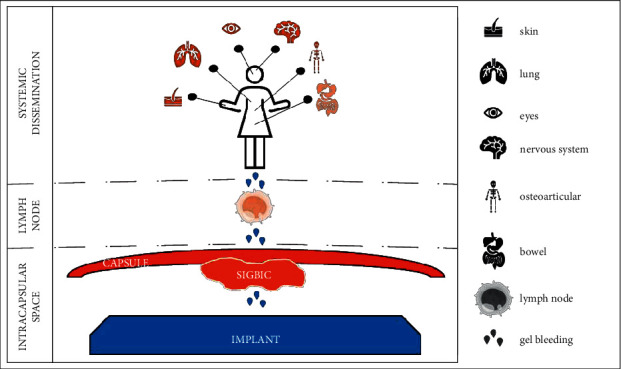
Example of silicone gel bleeding. The silicone will form the granuloma. As the implant is a continuous silicone generator, the silicone corpuscles can reach the pericapsular space. After going to the pericapsular space, silicone can reach the sentinel lymph node and later reach distant organs.

Silicone, especially with low weight, can also cross the fibrous capsule barrier, reach the pericapsular tissue, or migrate to distant organs, such as the intestine, ovary, and liver. In these cases, when there is a new inflammatory reaction of the fibrous capsule, the activated inflammatory process could target these distant organs. This activation usually determines clinical symptoms according to the affected organ, such as joint pain, skin eczema, and xerostomia [17] (Figure 2).

As the antigen generator is the silicone implant that permanently releases the silicone corpuscles, the clinical manifestations are evolutionary, with remitting and recurrent episodes.

## 4. Silicone Granuloma Diagnosis

Despite reports in the literature demonstrating the presence of silicone granuloma in surgical capsules and target organs since the 1970s, there was no diagnostic method or criteria for performing the diagnosis [18–20].

In 2016, we started a research protocol at the IBCC oncology. The protocol objective was to observe changes in silicone implants in patients referred for a breast MRI scan. The protocol prospectively evaluated approximately 1,000 patients with silicone breast implants [14]. All patients with silicone implants who showed changes in the MRI exam were referred for additional studies with ultrasound, biopsy, and surgery, as clinically recommended. We also correlated the imaging results with the pathology and clinical symptoms reported by the patients.

Early in the research, our main focus was to determine the prevalence of BIA-ALCL. We had a patient with an atypical intracapsular mass in the first cases, vascularized on ultrasound and magnetic resonance imaging. The patient underwent a percutaneous biopsy of the mass with the diagnosis of capsular contracture. The image was incompatible with a trivial capsular contracture. We asked the pathologist to search for free silicone in the biopsy specimens. After the second-look analysis, silicone granuloma was diagnosed, with giant cells and material refracting to polarized light in the microscopy analysis. We describe the findings as silicone-induced granuloma of breast implant capsules (SIGBIC) [21].

When the diagnosis was suspected of SIGBIC, we observed that two presentations were more prevalent on histology: intracellular silicone and extracellular silicone. The extracellular silicone would be the granuloma, a sequel of the inflammatory process with fibrosis, lymphocytes, and giant cells. Intracellular silicone represents the most acute phase, with foamy histiocytes and T lymphocyte dominance. The intracellular silicone is related to intracapsular collection, erroneously called a late seroma. We should void the late seroma descriptor due to the collection's high cellularity [22].

Pathologists need to describe histological findings related to silicone granuloma for accurate prevalence statistics. With the description, we should have the true prevalence of the event.

## 5. Imaging Diagnosis

**Figure 3:**
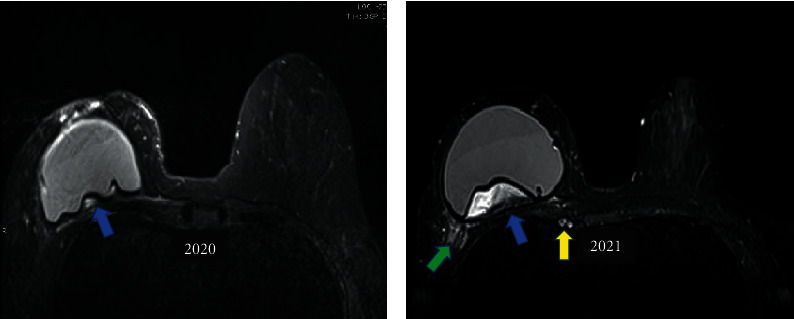
(a and b). Mass with a high signal on T2^*∗∗*^. Heterogeneous intracapsular tissue with a heterogeneous high signal is seen on the T2-weighted sequence^*∗∗*^ (blue arrow). Follow-up of silicone granuloma, 2020 (a) and 2021 (b). In the evolutive control, signs of pericapsular inflammation are also observed (green arrow).

**Figure 4:**
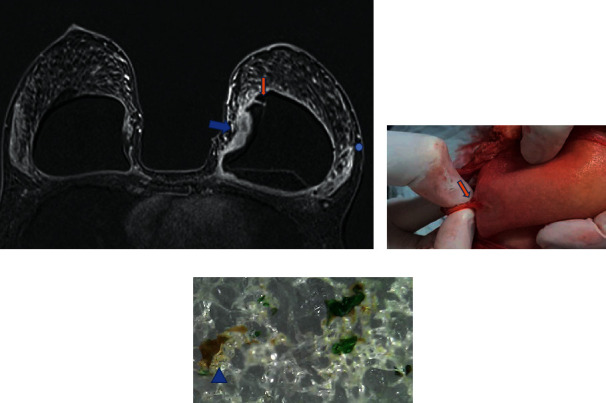
(a–c). Late contrast enhancement mass and a black-drop sign in a patient with polyurethane implant for 7 years. In the dynamic postcontrast T1-weighted sequence, the formation of an intracapsular mass (blue arrow) associated with a focus of marked low signal (black-drop sign) indicated by the blue circle. Macroscopy (b) shows intact implant with neovascularization in the implant surface (orange arrow). Microscopy of the implant surface (c) shows degradation of the surface, with fat inside the implant (blue triangle) and color change of the cohesive gel.

In our studies, we suggest three magnetic resonance imaging (MRI) diagnostic criteria for silicone granuloma: (1) intracapsular masses with a high signal in the T2^*∗∗*^ sequence (Figure 3); (2) delayed contrast enhancement in dynamic phases (Figure 4); (3) signal black-drop sign (Figure 4). When all three criteria are met, the diagnosis of SIGBIC can be made. As the diagnosis criteria are quite restrictive, we did not observe any false positive results of SIGBIC in complementary percutaneous biopsies or surgical specimens [23].

The high signal on T2 represents a high fluid content of the lesion, which is often misinterpreted as intracapsular seroma. On the other hand, late contrast enhancement means the low vascularization of the mass due to fibrous capsule protection. Finally, the black-drop sign is a granuloma characterized by marked hypointense foci in all sequences in the fibrous capsule. These signs are unequivocal in determining silicone leakage.

**Figure 5:**
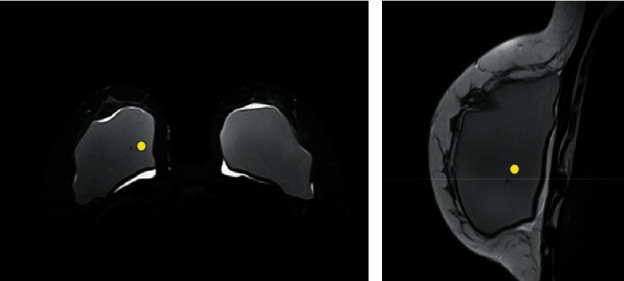
(a and b). Sign of permeability change of the implant surface (“water-droplet”) market with the yellow circle. This sign corresponds to the change of the selective permeability property of the implant shells. Axial STIR sequence (a) and sagittal proton-density sequence (b). Intracapsular collection is also observed.

Another sign described in our studies related to an implant surface permeability change is an abnormal focus inside the implant. The water droplet sign is water droplets inside the implant. When there is a permeability change in the implant surface, there is water flow between the inner and outer space of the implant (Figure 5). The liquid serves as a carrier for silicone particles. Implant color change from transparent to opaque (“clear to cloudy”) is reported in this situation [24, 25].

The average appearance of SIGBIC in patients undergoing MRI in our protocol occurs between 7 and 8 years after implant placement [14].

**Figure 6:**
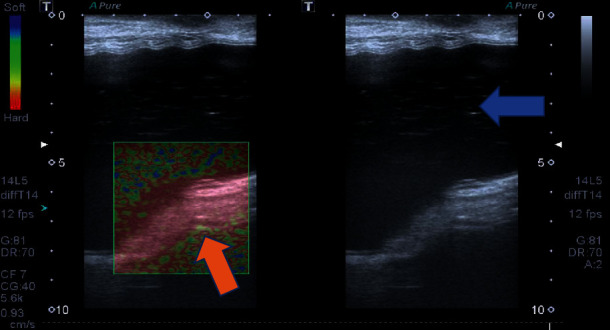
A 45-year-old patient with silicone implants for 7 years. Breast ultrasonography shows echotexture changes in the silicone implant contents (blue arrow) and an intracapsular mass associated with snowstorm artifacts, hard at elastography imaging compatible with silicone-induced granuloma of the breast implant capsule.

**Figure 7:**
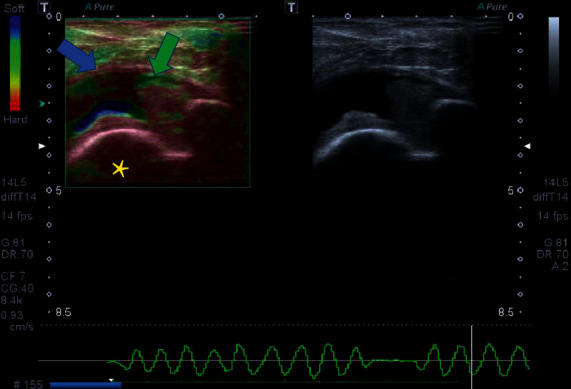
A 34-year-old patient with silicone implants for 5 years. Breast ultrasonography shows thickening of the breast implant capsule, associated with a granuloma in focal areas (green arrow). Intracapsular collection and intracapsular mass are also observed (blue arrow). The fluid collection is soft at elastography. The yellow asterisk shows the breast implant.

**Figure 8:**
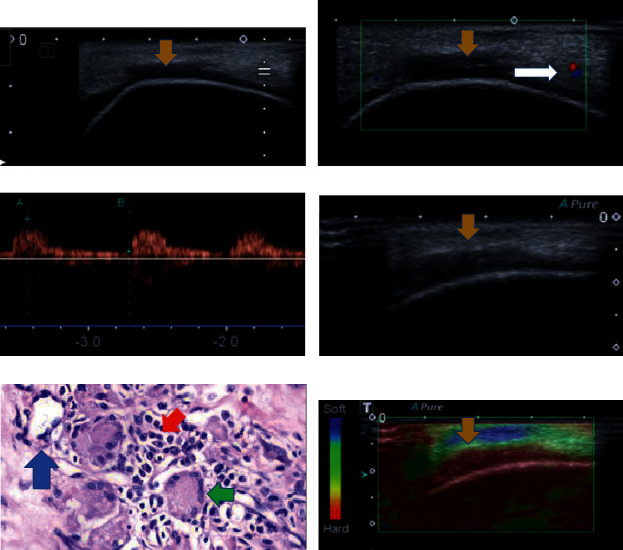
(a–f). A 34-year-old patient with silicone implants for 8 years. Breast ultrasonography shows thickening of the breast implant capsule, associated with pericapsular edema (yellow arrow). Figures b and c show increase in vascularity at the inflammatory site at collor (a) and spectral (b) images. Figures d and f show the hard pattern of the diseased capsule at elastography. The histology of the area shows inflammatory cells as lymphocytes (red arrow) and foamy histiocytes (green arrow) associated with silicone corpuscles (blue arrow).

The main ultrasound characteristics of SIGBIC are changes in the internal texture of the silicone implant indicating a chemical reaction, intracapsular mass with the snowstorm artifact and low vascularity at Doppler scan, fibrous capsule thickening, and intracapsular collection. Some extracapsular findings could be associated pericapsular edema with increasing vascularity, which is always found in the acute complicated phase. The elastography technique could help to evaluate the stiffness of the lesion (Figures 6–8).

**Figure 9:**
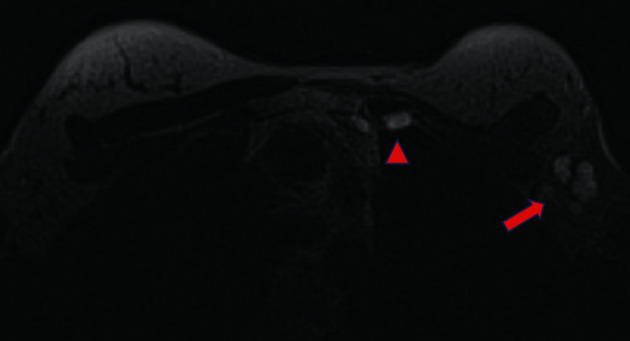
Example of silicone migration in silicone sensitive sequence to the axillary lymph node (red arrow) and internal thoracic (red triangle).

When the fibrous capsule barrier is impaired, a pericapsular enhancement inferring inflammatory process denotes the extra-capsular environment extension. There is often pericapsular intramammary lymph node enlargement with silicone, either in the internal thoracic or axillary chains (Figure 9).

**Figure 10:**
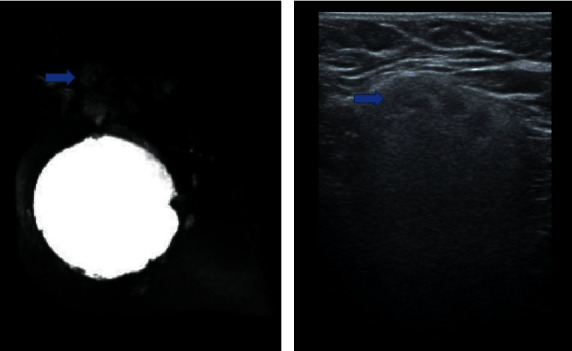
(a and b). 62-year-old women with silicone for 11 years. The MRI (a) shows intact silicone implant in the right breast associated with siliconomas in axillary lymph nodes. The axillary ultrasound (b) confirms the siliconoma as a mass with snowstorm artefact.

Ultrasonography is also useful to evaluate silicone migration to the axillary lymph node, where snowstorm artifacts will be present (Figure 10).

## 6. Relevance of Silicone Extravasation Diagnosis

Our study showed that many of our patients had clinical signs and complaints common to patients who reported breast implant illness (BII), a new disease controversial in the medical literature. Despite being questioned by the scientific forums, the FDA recognized BII as a complication of silicone implants in September 2020 [26–28]. Currently, the debate regarding BII has been spread across conventional and social media groups worldwide.

**Figure 11:**
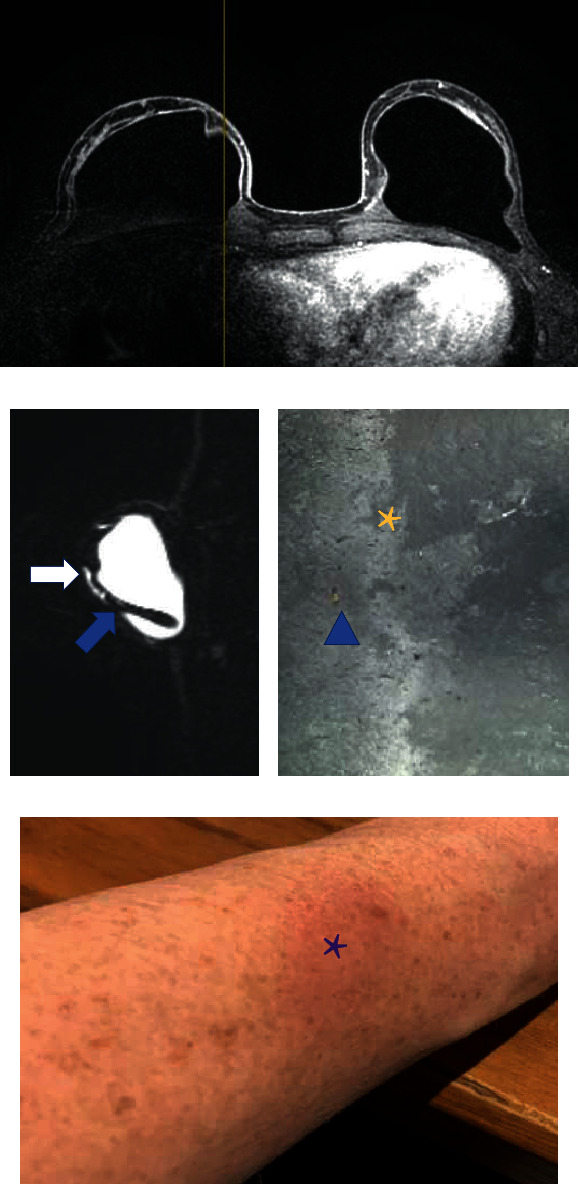
(a–c) Postcontrast sequence shows capsular contracture and focal thickness of the fibrous capsule (a). Silicone sensitive sequence (b) shows the granuloma and extracapsular silicone material in the pericapsular space. Microscopy of the implant (c) shows surface degradation in a smooth surface implant (yellow asterisk). There is also fat inside the implant. The patient also shows eczemas in the body, exemplifying the eczema in the right arm with the purple asterisk (d).

**Figure 12:**
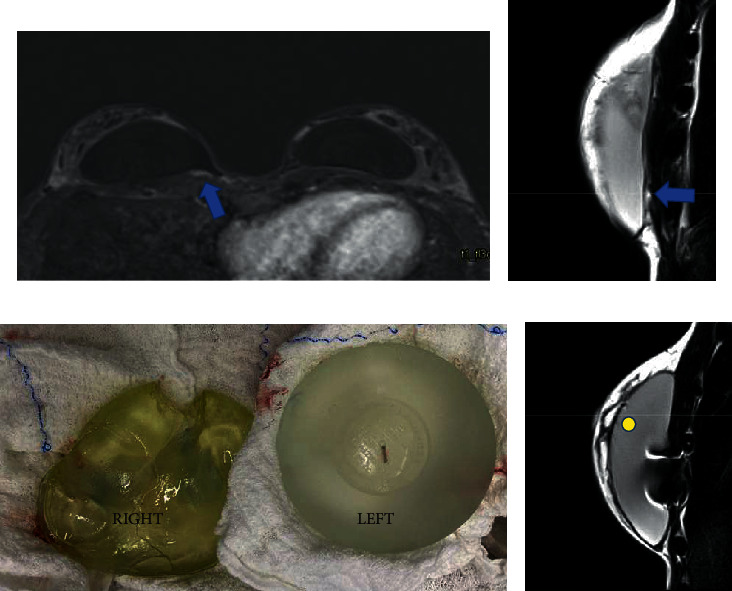
(a–d). A 37-year-old woman with nanotextured implant for 2 years, with BII complaints. Silicone granuloma in the right breast in postcontrast sequence (a) and proton-density (b) market with the blue arrow. The explant product confirming the MR findings, where the right implant shows surface degradation and color change (c). Figure D shows the radiofrequency identification artifacts and the water-droplet signal (yellow circle).

The relevance of SIGBIC diagnosis would be a diagnostic marker for silicone leakage in intact breast implants. In patients with symptoms of silicone disease, without clinical or laboratory diagnoses, the MRI findings could support implant degradation. On average, the implants have a useful life of 10 years according to the industry's recommendations (Figures 11 and 12).

Notably, SIGBIC can be found in any implant, saline or silicone-filled, due to silicone in the elastomer of the external shell. It can be seen on smooth surfaces, as well as on nanotextured and textured surfaces. In our experience, complications are more qualitative than quantitative and are independent of the amount of extravasated silicone. We thus observed the prevalence of changes in patients with a history of autoimmune diseases, such as psoriasis, rheumatoid arthritis, and Sjogren's syndrome [29].

## 7. Additional Findings to Silicone Bleeding

**Figure 13:**
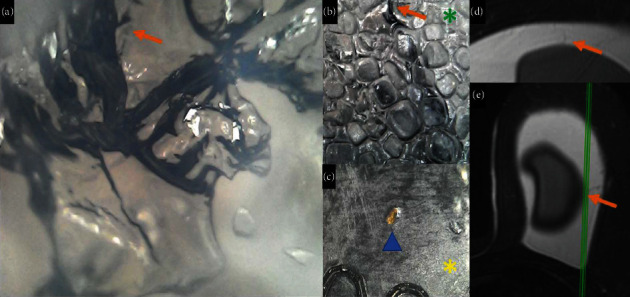
(a–e). Patients with textured breast implants with BIA-ALCL diagnosis presenting a sudden volumetric increase in the left breast. Microscopy shows neovascularization inside the implant in the orange arrow (a and b). The green arrow shows the pedicle in the textured surface (green asterisk), and in the smooth surface, there are fat infiltration (blue triangle) and surface degradation (yellow asterisk) in figure c (c). In the SPIR sequence, it is possible to see vascularization from the capsule to the implant (d and e) in the orange arrow.

We published scientific articles reporting SIGBIC in patients with clinical symptoms of BII, BIA-ALCL, aseptic pericapsular mastitis, and benign and malignant pericapsular neoplasms [30, 31]. Among the malignant neoplasms, undifferentiated and epithelioid carcinomas have been reported [32–35]. Our results follow those described in recent literature [14] (Figure 13).

In all cases, common findings are SIGBIC by imaging and intracellular and extracellular silicone with histology, with an essential monoclonal predominance of T lymphocytes.

## 8. Discussion

Breast implant illness is a novel disease reported by patients, especially in social media networks, where they have described common nonspecific symptoms that could affect all their organs and systems. Because of the nonspecific symptoms and the frequency of the same symptoms in the general population, these patients' complaints are often not valorized by clinical doctors. Currently, many articles have in fact assessed BII to be a myth.

However, in clinical practice, we observed a significant improvement in patients' health after their diseased fibrous capsule and the degraded implant were explained. The healing process is slow and progressive.

Since 2006, we have had millions of implant surgeries for aesthetic or reparative purposes with the end of the silicone implant moratorium worldwide. Since 2020, the FDA has added a black box recommendation based on possible issues regarding silicone breast implants. As such, the FDA advises that silicone should be replaced after ten years. The potential complications regarding such implants include BIA-ALCL, capsular contractures, gel bleeding, and systemic symptoms related to BII.

Unfortunately, there is little evidence reported in the literature on diagnosing, managing, and following up on patients with breast implants.

Overall, in this review, we discuss the current knowledge regarding breast implant illness and emphasize the crucial role of the radiologist in diagnosing this curious and novel man-made disease.

## Data Availability

The data used to support the findings of this study are available from the corresponding author upon request.

## Abbreviations

BIA-ALCL: Breast implant-associated anaplastic large cell lymphoma
ASIA: Autoimmune/inflammatory syndrome induced by adjuvants
FDA: Food and drug administration
PDMS: Polydimethylsiloxanes
SIGBIC: Silicone-induced granuloma of breast implant capsules
MRI: Magnetic resonance
BII: Breast implant illness.

## Additional Points

*Keypoints.* Silicone granulomas could be diagnosed by MRI. Radiologists should be versed in the pathophysiology of silicone breast implant disorders.
